# Copy number variant scan in more than four thousand Holstein cows bred in Lombardy, Italy

**DOI:** 10.1371/journal.pone.0303044

**Published:** 2024-05-21

**Authors:** Andrea Delledonne, Chiara Punturiero, Carlotta Ferrari, Francesca Bernini, Raffaella Milanesi, Alessandro Bagnato, Maria G. Strillacci

**Affiliations:** Department of Veterinary Medicine and Animal Science, Università degli Studi di Milano, Lodi, Italy; West Bengal University of Animal and Fishery Sciences, INDIA

## Abstract

Copy Number Variants (CNV) are modifications affecting the genome sequence of DNA, for instance, they can be duplications or deletions of a considerable number of base pairs (i.e., greater than 1000 bp and up to millions of bp). Their impact on the variation of the phenotypic traits has been widely demonstrated. In addition, CNVs are a class of markers useful to identify the genetic biodiversity among populations related to adaptation to the environment. The aim of this study was to detect CNVs in more than four thousand Holstein cows, using information derived by a genotyping done with the GGP (GeneSeek Genomic Profiler) bovine 100K SNP chip. To detect CNV the SVS 8.9 software was used, then CNV regions (CNVRs) were detected. A total of 123,814 CNVs (4,150 non redundant) were called and aggregated into 1,397 CNVRs. The PCA results obtained using the CNVs information, showed that there is some variability among animals. For many genes annotated within the CNVRs, the role in immune response is well known, as well as their association with important and economic traits object of selection in Holstein, such as milk production and quality, udder conformation and body morphology. Comparison with reference revealed unique CNVRs of the Holstein breed, and others in common with Jersey and Brown. The information regarding CNVs represents a valuable resource to understand how this class of markers may improve the accuracy in prediction of genomic value, nowadays solely based on SNPs markers.

## Introduction

For millennia, humans have established a profound relationship with cattle domesticating them to exploit their resources, obtain food as milk, and meat, and meet various needs [1]. Since the 20^th^ century, the selection to improve production traits in animal species, such as the Holstein cattle breed, represents a fundamental step in the development of modern animal husbandry. The Holstein breed, nowadays worldwide recognized for its milk production, has undergone a strong selection effort aimed at improving milk yield, quality, and in the last two decades in enhancing overall functionality and health [2]. In recent years, the evolution of nanotechnology made available the SNP genotyping platforms that made possible the genomic selection revolution in cattle breeding theorized by Meuwissen et al. [3]. The utilization of SNP chips in genotyping has proven to be a potent tool in animal selection, empowering breeders to make well-informed decisions based on the collective genetic information [4]. SNP genotyping data also enable the detection of Copy Number Variants (CNVs) through the computation of the Log R Ratio (LRR) and B Allele Frequency (BAF). LRR represents a normalized measures of the total signal intensity for two alleles of a SNP, and the B allele frequency (BAF), the one measuring the allelic intensity ratio at marker level [5]. The LRR and BAF facilitate the assessment of CNV status (loss vs gain, LRR; homozygote vs heterozygote, BAF). CNVs represent a category of genomic structural variants recognized to influence phenotypic diversity through the deletion (loss status) or duplication (gain status) of DNA segments, potentially affecting gene structure and regulating expression [6, 7]. These variations typically range in size from 1 kilobase (kb) to 5 megabase (Mb) [8].

The functional impact of CNVs has been studied across various animal species, highlighting their role in influencing a range of phenotypic traits [9–13]. The fact that CNVs affect a multitude of traits across different animal species underlines their role also in adaptive responses to various environmental conditions [14–17]. In several studies on Holstein cattle, CNVRs have been identified to impact economically important traits as milk production, residual feed intake, fertility and somatic cell score [18–21].

Although CNVRs cover a small part of bovine genome length (about 2–10%), as reported by [22], these structural variants can be integrated with SNP information in genomic prediction, offering new insights to explain complex traits and understand the proportion of missing heritability not explained by SNP.

Thus, taking into account all information related to Copy Number Variations (CNVs), the objectives of this study were to examine a substantial population comprising 4,282 Holstein cows from seven distinct farms in Italy, with the purpose of map CNVs across the autosomal genome. Additionally, within the more frequent CNVRs, the goal encompassed the annotation of genes and of quantitative trait loci (QTL) associated with relevant traits in this breed. To validate our findings, we conducted a comparative analysis both within and across different cattle breeds, drawing on insights from prior research studies.

## Materials and methods

### Animal sampling, genotyping and ethics statement

All cows of 7 herds of the Lombardy region were genotyped with the Illumina GGP Bovine 100K (GeneSeek®) from 2019 to 2023 for a total of 4,282 individuals. These 7 herds are representative of the possible farming systems and selection objectives of Holstien farmers: they in fact spans from a small family run farm (110 cows in lactation) with historically low selection, to a large farm with Automatic Milking System and with more than 3 decades of directional selection to improve production and functionality (about 550 lactating cows) and a medium size farm producing Parmigiano Reggiano cheese and thus, requiring specific nutritional practices (no silage) and selection for milk quality. Log R Ratio (LRR) available from the SNP chip processing were used to map CNVs. The quality assessment of LRR and the mapping of CNVs was performed with the Golden Helix Inc. SVS 8.9 software (SVS).

The sampling of individual was approved by the OPBA (*i*.*e*., Animal Welfare Organisation) of the University of Milan (Protocol number 160_2019), by Directive 2010/63/EU of the European Parliament and the Council of 22 September 2010, updating Directive 86/609/EEC on the protection of animals used for scientific purposes.

### Quality control of genotyping data

The quality assessment of LRR values was performed considering the Derivative Log Ratio Spread (DLRS) as described by Pinto et al. [23] and the GC Wave Factor (GCWF) [24], both affecting signal intensity and possible cause of bias in CNVs mapping. A total of 47 samples were excluded due to their high DLRS values, while other 135 samples were excluded because of the elevated GCWF values. The detection of CNVs was then conducted on a dataset of 4,100 samples.

### CNVs and CNVRs detection

CNVs detection was obtained on autosomes with SNPs mapped on the ARS UCD1.2 assembly reference genome. The detection was performed using the Copy Number Analysis Module (CNAM) of SVS by means of the univariate analysis based on LRR values. Default parameters for CNVs calling in CNAM were set as follows: i) a maximum of 100 segments per 10,000 markers; ii) a minimum of 3 markers per segment; iii) 2000 permutations per pair with a p-value cut-off of 0.005.

To identify animals with outliers CNVs frequencies and length, their distributions were analysed using QQ plots (R routine in ggplot2 library [25]). Outliers were identified as samples having CNV length greater than 7.5 Mbp. After the identification and exclusion of the individuals considered outliers (3,809 subjects were left), the individual frequency of gain and loss in relation to each sample mean CNVs length was plotted with the ggplot2 library of R.

Using the Bedtools -mergeBed command [26], CNVs that overlapped by at least one bp and were shared by a minimum of two animals were combined to generate CNV regions (CNVRs). Then, CNVRs were classified as gain, loss, or complex if comprising both deletions (loss) and duplications (gain). A CNV found in a single individual was classified as a singleton CNVR.

To be representative, only CNVRs shared by at least 2% of the population were selected for descriptive statistics as well as for downstream analyses.

The R package HandyCNV [27] was used to visualized the physical distribution of CNVRs on autosomes.

### Genes and QTL annotations

The genes list with official “gene name ID” was downloaded from NCBI online Database. Genes were then annotated within the detected CNVRs using the Bedtools “-intersectBed” command [26], while the QTL associated with the genes found in the CNVRs were identified thanks to the cattle QTL database (https://www.animalgenome.org/cgi-bin/QTLdb/BT/search) by gene name, using the “Search by associated gene” option of QTLdb.

The Cytoscape plugin ClueGo was used to identify potential biological connections among candidate genes identified in the CNVRs [28, 29]. The network construction relied on information from GO and KEGG database. This analysis utilized the bovine databases integrated into the ClueGO app. Only connections with a p-value lower than 0.05 were considered.

### Diversity at the population level

To study the diversity within the breed we recoded CNVs defining a CNVR for each cow as follows: i) ’1’ for loss state; ii) ’0’ for normal state; iii) ’2’ for gain state. We used the Past 4.03 software to perform a principal component analysis (PCA).

### Comparison with results from the literature

Our identified CNVRs were compared with the results reported in recent literature studies using the HandyCNV library of R-Studio software (compare_cnvr() function).

As reported in Table 3, two distinct comparisons were performed in order to validate Holstein specific CNVRs (comparison within breed), and to identify genomic regions shared by different breeds (comparison among breeds), i.e. Jersey (JER) and Brown Swiss (BSW). For studies with CNVRs using a different genome assembly from ARS-UCD1.2, the positions were remapped using the UCSC Lift Genome Annotations tool (https://genome.ucsc.edu/cgi-bin/hgLiftOver). A graphical visualization of overlapped CNVRs was realized through a Venn diagram built using an online tool (http://bioinformatics.psb.ugent.be/webtools/Venn/).

## Results

### CNVs and CNVRs detections

According to the number of CNVs per cow and their total length (sum of each CNV length), 291 samples were identified as outliers and subsequently removed to avoid the introduction of possible false positive CNVs; the final dataset comprising 123,814 CNVs was obtained in 3,809 cows (S1 Table); with a total of 4,150 non-redundant CNVs.

As reported in Table 1, CNVs have a maximum, minimum, and average length of 1,860,579, 1,005 and 86,166 bp, respectively. The frequency of loss CNVs doubles the frequency of gain CNVs and the mean length of losses (90,439.4) is longer than the mean length of gains (77,785.5).

**Table 1:** Descriptive statistics of identified CNVs.

N.	N. CNVs	N. Gain	N. Loss	Loss/Gain	Min-Max CNV per ID (mean)	Min-Max (mean) length*	Min-Max (mean) coverage per ID*
3,809​	123,814	41,556	82,258	1.98	13–51 (32.5)	1–1,860 (86)	947–7,483 (2,792)

*Value expressed in Mbp.

**Fig 1:**
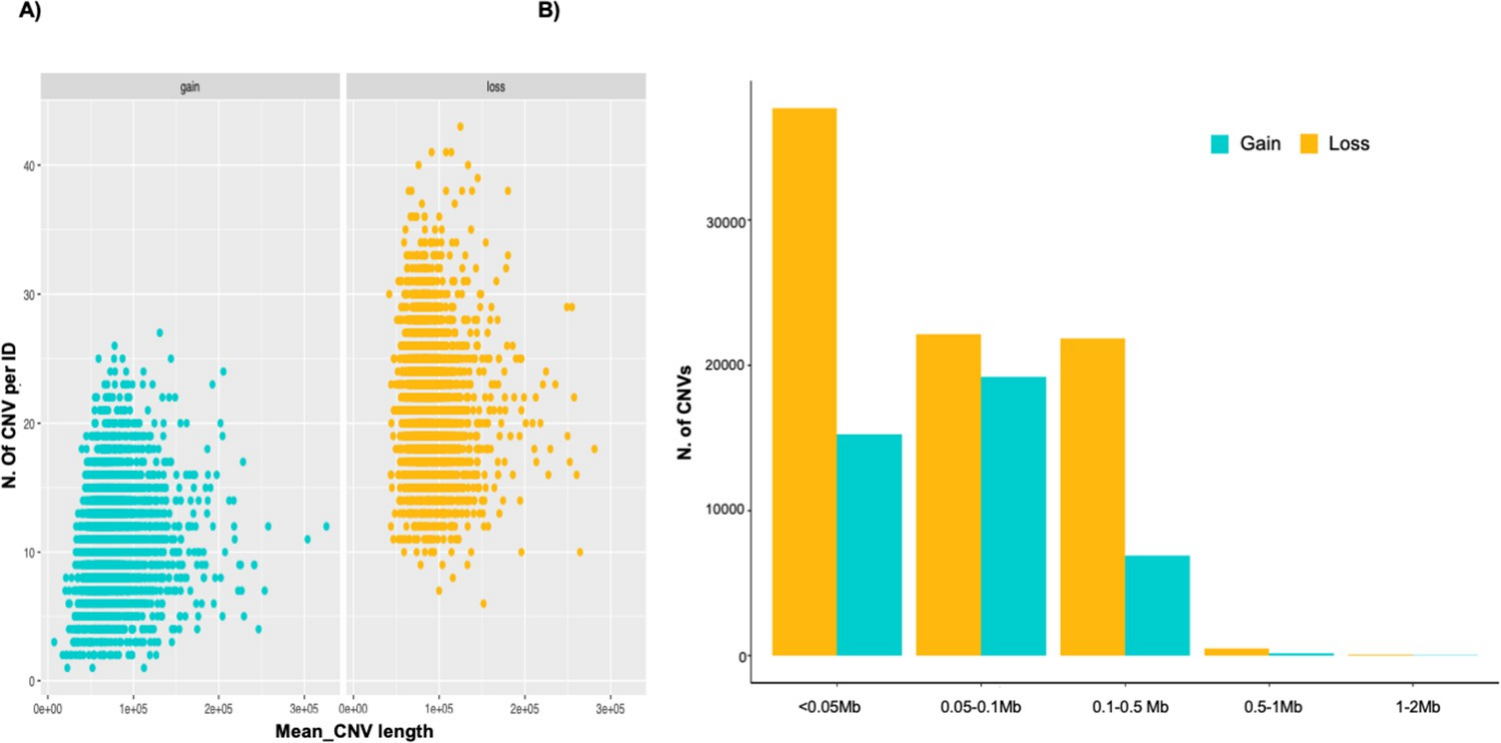
Summary of statistic for detected CNVs. A) Relationship between number and mean total length (bp) of CNVs identified in each sample by state (gain vs loss); B) Number of CNVs for five classes of length.

Fig 1A shows the different distribution of gain and loss CNVs according to the relationship between the CNV mean length and their number per samples. Furthermore, as shown in Fig 1B, the majority of CNVs falls into the first three classes of length. Over 30,000 loss state CNVs exhibited a length below 0.05, falling in the first length class. Conversely, the majority of gain CNVs had a length ranging between 0.05 and 1 Mb. The longest CNVs were low represented for both of CNV states.

**Fig 2:**
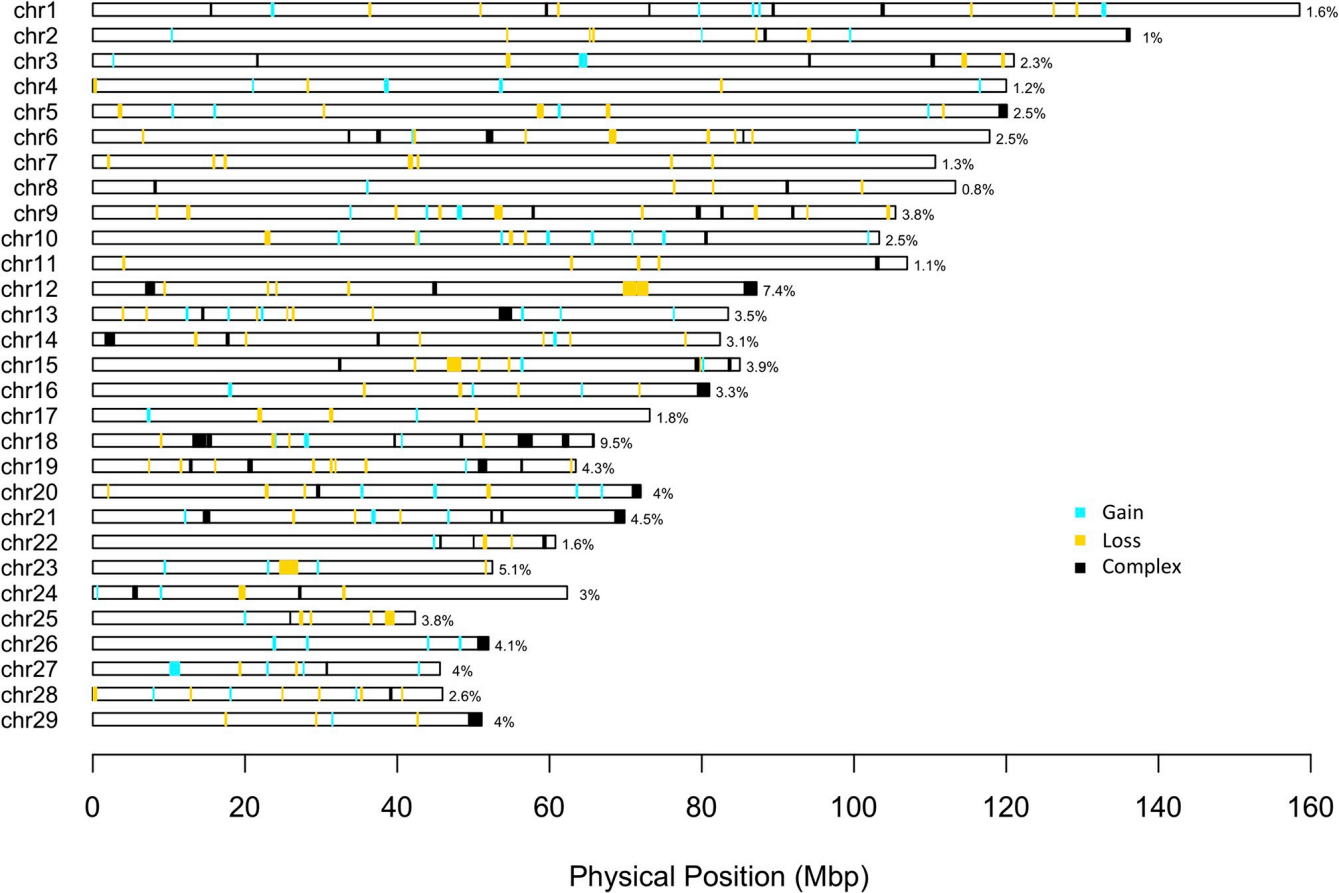
Physical distribution of the Copy Number Variants Regions (CNVRs) according to states (complex, gain and loss) on the *Bos taurus* ARS-UCD 1.2 assembly. Plotted CNVRs are those shared by at least 2% of individuals. Percentage values refer to the genomic proportion covered by CNVRs respect to the BTA length.

The 123,814 CNVs were aggregated into 1,397 CNVRs (Table 2 and S2 Table), covering 9.18% (228 Mbp) of the total autosomal length (2,489 Mbp). After removing singletons and CNVRs shared by less than 2% of the population, 267 CNVRs remained (CNVRs_2% in Table 2 and S2 Table): 76 in gain state, 129 in loss and 62 categorized as complex. CNVs in CNVR_2% are listed in the S2 Table. These latter CNVRs cover 2.92% of the autosomal genome length and their physical distribution on autosomes is shown according to their states in Fig 2. Values (%) on this graph represent the genomic proportion covered by CNVRs with respect to each chromosome length. CNVRs on chromosomes 12, 18 and 23 covered more than 5% of chromosomal length, 9.5%, 7.4% and 5.1% respectively, while all other chromosomes were impacted by a lower proportion of CNVRs. The CNVRs shared by the largest number of cows were on BTA 10 at 22,676,353 bp (n. 3,528 cows, loss) and on BTA 2 at 93,926,090 (n. 3,107 cows, loss). Instead, CNVRs shared by the lowest number of cows, i.e. 76 animals, were found in gain state within chromosome 20 (at 66,818,777 bp).

**Table 2:** Descriptive statistics of identified CNVRs.

CNVRs	Tot n. CNVRs	Tot n. Singleton	CNVRs State			CNVRs length			
			Loss	Gain	Complex		Min	Max	Mean
CNVRs	1,397	329	714	513	170		1,005	2,286,232	163,678
CNVRs_2%	267	-	129	76	62		1,716	2,286,232	272,307

S1 Fig shows the genome-wide distribution of the 267 CNVRs across the chromosomes together with the mean CNVRs coverage length. The maximum number of CNVRs are on BTA 1 and BTA 9. The mean CNVRs length is not uniform along all chromosomes, and the maximum mean CNVR length was on BTA 12 (717,015.8 bp).

**Fig 3:**
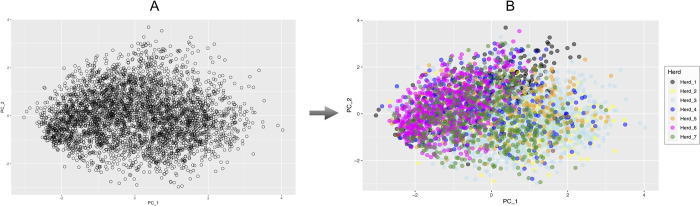
Principal component analysis results. A) Samples are coloured in black as unique Holstein breed; B) Samples are coloured according to the herds in which the cows were sampled.

Principal component analysis results (Fig 3A and 3B) depict the genetic variability in the 3,809 cows analyzed, according to the presence or absence of CNVs in the identified CNVRs, considering their state. Each point in the scatter plots represents an individual animal, coloured as unique population (Fig 3A) or taking into account the herd from which it was sampled (Fig 3B).

### Gene content and annotation

A total of 996 genes were annotated within 194 Holstein CNVRs (72.6% of the CNVRS_2%). Their functional classification, according to the David database, is reported in the S3 Table (recognized gene IDs = 942).

In S2 Fig (ClueGo network) it’s possible to observe the presence of five macro-groups of genes associated with the following categories: troponin complex, sensory perception of smell, nervous system process, tuberculosis, and MHC class II protein complex. The KEGG pathway comprising the majority of genes is the one connected to tuberculosis, the same result has been obtained with David analysis.

**Fig 4:**
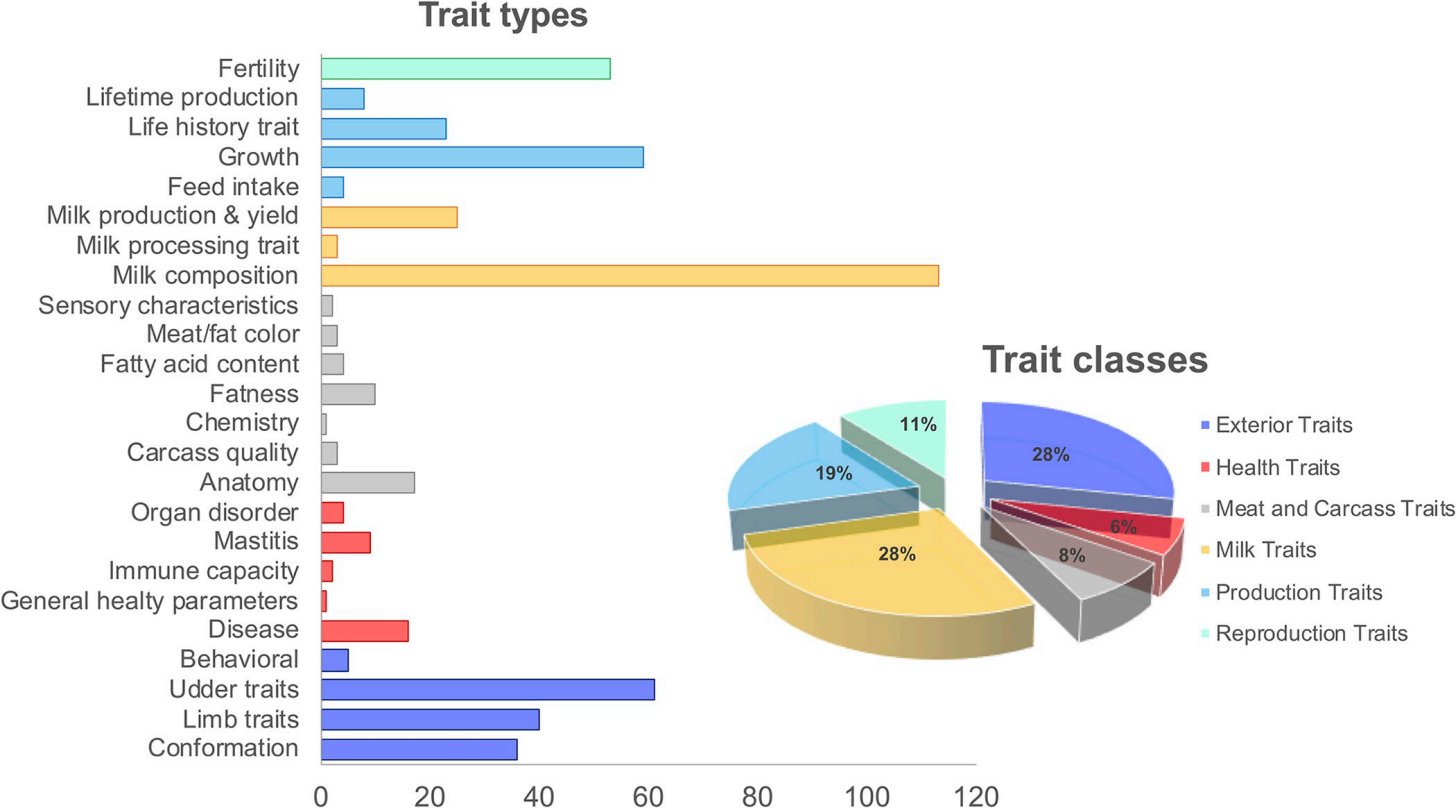
Graphical representation of QTL_terms (Trait types and Trait classes) associated with genes mapped in CNVRs. Colours of Trait types corresponded to the ones in Trait classes.

After consulting the Cattle QTLdb, 142 genes were associated with a total of 122 different “Trait Name”, grouped into 24 “Trait Types” corresponding to 6 “Trait Classes” (Exterior, Healthy, Meat and Carcass, Milk, Production, and Reproduction Traits), in concordance with the database nomenclature (Fig 4). As Fig 4 shows, the most of traits associated with the genes annotated in the CNVRs are related to the phenotypes for which the Holstein population has been selected for years.

### Comparison with references

**Fig 5:**
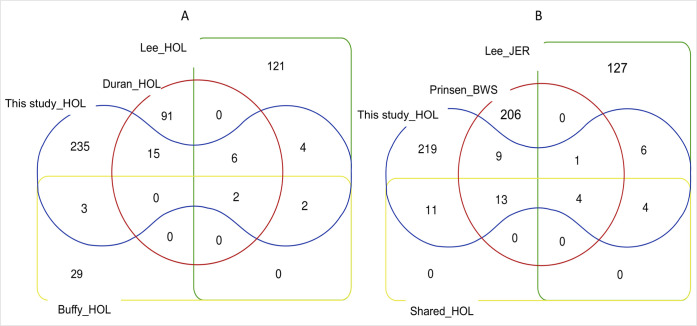
Comparison of CNVRs identified in different Holstein populations (A) and in others two breeds (B). Shared_HOL are those CNVRs (n.32) identified in at least two studies (part A of this Fig).

CNVRs here identified were compared with those identified in three other Holstein populations (comparison within breed) and in two different breeds (comparison among breed; one dairy cattle–Jersey; one dual-purpose cattle–Brown Swiss) (Table 3 and Fig 5 and S2 Fig and S4 Table). As reported in Table 3, the minimum and the maximum number of overlapping regions were 7 and 27, respectively.

**Table 3:** Comparison with literature.

Platform	Software	Breed (N. of IDs)	Reference Genome	N. CNVRs^a^	N. overlapped CNVRs^b^	Overlapping length (bp)	Ref
Comparison within breed							
Illumina HD; 50K; GGP150K	PennCNV	Holstein (96)	ARS-UCD1.2	36	7 (2.6%; 19.4%;)	1,239,370	[30]
Ilumina HD	PennCNV	Holstein (315)	ARS-UCD1.2	135	14 (5.2%; 10.4%)	1,374,082	[31]
Illumina HD	CNAM (SVS)	Holstein (242)	UMD3.1 remapped	112	23 (8.6%; 20.5%)	12,017,083	[21]
Comparison among breeds with different aptitude							
Illumina HD	PennCNV	Jersey (107)	ARS-UCD1.2	142	15 (5.6%; 10.5%)	1,915,749	[31]
Illumina HD	CNAM (SVS)	Brown Swiss (1,116)	UMD3.1 remapped	233	27 (8.6%; 11.6%)	10,651,728	[32]

^a^When remapped, this number refers to the CNVRs resulting after the positions remapping

^b^Proportion of overlapping: calculated as n. overlapped CNVRs/n. CNVR in this study; n. overlapped CNVRs/n. CNVR in other study.

The 48 CNVRs resulted overlapping regions (S4 Table) included 32 regions identified in others Holstein samples, i.e. CNVRs mapped in at least two studies (shared_HOL) as shown in Fig 5A. When the comparison was performed with the JER and BSW cattle, the 32 shared_HOL regions in Fig 5B, resulted in 11 Holstein proprietary CNVRs and 4 ones found in all breeds. As in Fig 5B, the BSW breed shared the largest number of overlapping regions CNVRs. The total overlapping CNVR length was similar for those studies in which CNVs were identified with the same software (< 2 Mb–PennCNV and > 10 Mb–SVS, Table 3).

## Discussion

In the literature there are several studies investigating genetic variability of Holstein’ population using SNPs, and to increase knowledge on this breed, a large set of Italian Holstein cows has been inhere analyzed through CNVs detection. CNVs, a class of structural variation, can inform about population variability and are known to occur in the genome in response to environmental stressors, including positive selection, as a consequence of farming strategies [33].

This study, based on a medium density SNP chip, i.e. the Illumina GGP Bovine 100K, allowed the identification of a high number of CNVs in a substantial number of Holstein cows. The number of CNVs per sample (32, on average), is relatively higher compared to studies that rely on non-dense SNP chips, but lower compared to studies that rely on dense SNP chips or use sequences to call CNVs [34–36]. As reported in the majority of CNV mapping studies performed with Illumina SNP chips, the number of deletions calls was approximately 1.98 more recurrent than duplications [21, 31, 37]. The mean length of deletion calls inhere (90,439.4 bp) is bigger than the mean length of found gains (77,785.5 bp). Interestingly Lee *et al*. [31], using the Illumina BovineHD BeadChip, found that duplications are longer then deletions.

Overlapping CNVs resulted in 1,397 CNVRs covering 9.18% of the cattle genome. This value is much higher than the ones reported in the literature for Holsteins, which range from 0.5% to 2.8% [31, 38], but in line with the coverage found by Butty et al. [30], depending on the density of the SNP chip and the detection algorithm used [30, 39]. When CNV regions shared by at least 2% of the population were selected, the percentage of genome covered by CNVRs decreased (2.9% of the autosomal genome length, Fig 2), a value similar to those reported by other authors [31, 38].

As shown in S1 Fig, CNVRs are not uniformly distributed on the autosomes, and the distribution of CNVRs according to their length class (Fig 1C) shows that the majority are short to medium in length and only a few are observed in the long classes, consistently with previous findings [31].

To visualize the genomic variability related to CNVs detected in our study population, we performed a Principal Component Analysis and the results in Fig 3A, at first glance, show that all animals are spread in the graph without any clustering tendency.

The homogeneous grouping in this study appears to be related to the fact that all the cows, although bred on different farms, undergone similar intensive farming system. Nevertheless, the genetic selection performed by the farmers seems to produce an effect: when the grouping animals by herd (Fig 3B) a slight clustering can be observed, mainly for animals in Herd_6 (magenta colour). In Herd_6, mating plans have been based on bulls from a unique AI center for years, while all other herds use sires from different semen providers [40]. When the gain/loss ratio was calculated in each herd to explain our findings, it was equal to 0.40 in Herd_6 (this value correspond to a loss/gain ratio = 2.40) and up to 0.49 in all the others herds (maximum value was 0.70 in Herd_5; loss/gain ratio = 1.41). The lower proportion of gain CNVs found in Herd_6 may be linked to the highest number of daughters for sire in Herd_6, with a reduction of variability in specific genomic regions. The lower number of common bulls across all herds (as reported by Punturiero et al. [40]) can explain the cows’ distribution of Herd_6 respect to the ones belong to all other farms. In Herd_5, the number of daughters per sire is one of the lower.

### Gene content and annotation

According to the David database (S3 Table), the genes annotated within the CNVRs were classified in 91 Go-Terms. The KEGG pathway analysis revealed that among the genes under analysis 56 are mainly represented in the pathway of immune system, namely, in the classes “Tuberculosis” and “Staphylococcus aureus infection”, and in the pathway of thermogenesis. Disease resistance (or susceptibility) is a complex trait and interestingly it could be affected by genomic variations, as found by different authors reporting a substantial immune gene enhancement within CNV regions [21, 41–43].

The network constructed with ClueGO (S2 Fig) aligns with the results found with the David analysis. It’s possible to see genes connected to different GO categories linked to nervous system, troponin complex, sensory perception of smell, nervous system process, together with the KEGG category of susceptibility to tuberculosis. Some genes are connected with more than one category, for example BOLA genes.

Variation in gene copy number leads to phenotypic variation among animals. After consulting the AnimalQTLdb for cattle we grouped the QTL in 24 trait types. As listed in the S3 Table and showed in Fig 4, the most common trait type is milk composition, for which 102 QTL were found. This result is in line with the expectations, being the animals part of commercial farms that sell milk for the dairy industry. Milk composition, together with udder conformation, fertility, and growth (more representative trait types in Fig 4) are all object traits of selection for high-productive breeds, such as the Holstein.

### Noteworthy CNVRs and comparison with references

Nine CNVRs resulted over-represented due to a high number (> 2,000) of CNV defining these regions: 4 CNVRs do not harbor genes, and most of them are in loss state. The only duplication region is the cnvr_234 identified on BTA 25 (in 2,400 cows) (S2 Table). In this CNVR, map the EEF2K and POLR3E genes that are involved in the cellular response to oxidative stress [44] and the host innate immune defense against viruses [45], respectively. Even for the genes mapped in the cnvr_024 (3,107 cows) on BTA 2 (PARD3B, NRP2) a roles in immune response was reported [46, 47]. Finally, the cnvr_069 located on BTA 7 (2,156 cows) overlaps the CNVR20 (complex state) identified by [30]. This region harbors five genes belonging to the family 2 of olfactory receptor genes (OR). CNVs are frequently found within OR genes and this variability may contribute to individual or breed-specific differences in olfactory capacity [48], which is also associated with feed intake and efficiency [49]. This aligns with the findings in our research; indeed, conducting gene ontology analysis with ClueGO (S2 Fig) yielded results for 35 genes in a copy number variation state linked with the following functional categories: sensory perception of smell, detection of stimulus involved in sensory perception, detection of chemical stimulus involved in sensory perception, olfactory perception activity and sensory perception of chemical stimulus. Nonetheless, these results only contribute to a small portion of our understanding given the size and complexity of this gene family comprising more than 1,000 known OR genes.

Regarding the comparison with references, as reported in Table 4, among the 267 CNVRs, 11 overlapped with the ones identified only in Holstein populations and 4 in all the considered breeds (Holstein, Jersey, and Brown). It is important to note that the size of the CNVRs identified in this study decrease after comparison (we reported only regions perfectly overlapping). This is particular evident for cnvr_225, splitted in two small regions as listed in Table 4. The entire cnvr_225 harbour genes belonging to the BOLA family, a well known gene implicated in host immune response. In the cnvr_133, located on BTA 13 (both in loss and complex states, according to breeds, see S4 Table), lied the SIRPB1 gene, also involved in the immune response [50].

**Table 4:** CNVRs in common between our study and the ones found in Holstein and in different cattle breeds.

CNVR_ID this study	Chr	Start	End	State	Genes	QTL
**Common CNVRs (HOL, JER, BSW)**						
cnvr_075	8	76336567	76348332	loss		
cnvr_121	12	71701903	71765886	complex		
cnvr_133	13	53463194	53511604	complex	SIRPB1	SIRPB1: Milk protein percentage (QTL: 174904)
cnvr_225	23	25953514	26064642	complex		
cnvr_225	23	26113327	26350925	complex		
**Only HOL CNVRs**						
cnvr_034	3	119662571	119718948	loss	COPS9, OTOS	
cnvr_035	4	182210	217902	loss		
cnvr_055	6	37695352	37736960	complex		
cnvr_058	6	51884459	52200066	complex		
cnvr_068	7	17374656	17409367	complex		
cnvr_072	7	81385397	81392696	loss		
cnvr_137	14	1645654	2064157	complex	LY6D, LYNX1, LYPD2, SLURP1,THEM6, PSCA, TSNARE1, ARC, ADGRB1, JRK	LY6D: Milk fat percentage (QTL:33308; 166962; 161706), Milk protein percentage (QTL:161824)
cnvr_176	18	27856333	28303561	complex		
cnvr_181	18	57234258	57254890	complex		
cnvr_216	21	69040055	69788216	complex	C21H14orf180, TMEM179, INF2, ADSSL1, SIVA1, AKT1, ZBTB42, CEP170B, PLD4, AHNAK2, CLBA1, CDCA4, GPR132, JAG2, NUDT14, BRF1, BTBD6, PACS2, TEX22, MTA1, CRIP2, CRIP1, TEDC1, TMEM121	AKT1: Bovine respiratory disease susceptibility (QTL: 160320; 160321); BRF1: Conception rate (QTL: 123998)
cnvr_245	26	50598736	51990348	complex	KNDC1, ADGRA1, CFAP46, NKX6-2, INPP5A, BNIP3, JAKMIP3, DPYSL4, STK32C, LRRC27, PWWP2B	

Across the identified CNVRs proper of the Holstein cows, a wider variability in the regions state can be observed, more than 70% are in fact in complex state. Only 4 CNVRs harbour genes. Among them, cnvr_137 contains genes such as LY6D, LYNX1, LYPD2, SLURP1,THEM6, PSCA, TSNARE1, and ARC associated to clinical mastitis in US Holstein dairy cows [51]. While the cnvr_245 includes the BNIP3 gene, that plays a critical role in inducting autophagy during heat stress and was associated with the immune response phenotype [52]. The same region partially overlaps the CNVR_1549_P (the region comprising the JAKMIP3, DPYSL4, STK32C, LRRC27, PWWP2B) resulted associated with clinical mastitis in Mexican Holstein Cattle [21].

## Conclusions

The study provides novel insights into CNVs mapped within the Italian Holstein cows. To date, this is the only study that conducted a CNV analysis on such a large number of animals within this breed. Based on CNVs, the Principal Component Analysis (PCA) revealed a homogeneous distribution of cows, indicating a shared effect of the intensive farming system on these animals. The slight clustering observed among cows from the same farm implies that genetic selection may influence CNV distribution, underscoring the potential impact of selective breeding practices.

The functional analysis of genes annotated in the more common CNVRs revealed biological mechanism related to immune resistance to infection and adaptability. QTL linked with the main traits object of directional selection overlapped with many CNVRs here identified. Genes involved in immune response and defense against oxidative stress were identified within CNVRs, suggesting that genetic variability could affect the animals’ ability to respond to environmental stressors.

The analysis of CNVs not only provides an additional dimension of genetic information, but also represents a valuable resource to optimise (new prespective) genomic selection in a more complete and accurate way.

## Supporting information

S1 Table CNVs identified in Holstein breed. (XLSX)

S2 Table List of the total CNVR (sheet_1); CNVRs identified in at least 2% of cows (sheet_2), ad list of CNV defining CNVRs identified in at least 2% of cows. (XLSX)

S3 Table Gene functional annotation from David database. (XLSX)

S4 Table CNVRs comparison with references. (XLSX)

S1 Fig Graphical representation of CNVRs number and mean CNVR coverage length on autosomes. (TIF)

S2 Fig ClueGo network of genes annotated in CNVRs identified in at least 2% of cows. (TIF)
