# Supplementary figures and images for: Copy number variant scan in more than four thousand Holstein cows bred in Lombardy, Italy
Source: PLoS One. 2024 May 21;19(5):e0303044. doi: 10.1371/journal.pone.0303044 (PMC11108207; doi:10.1371/journal.pone.0303044)

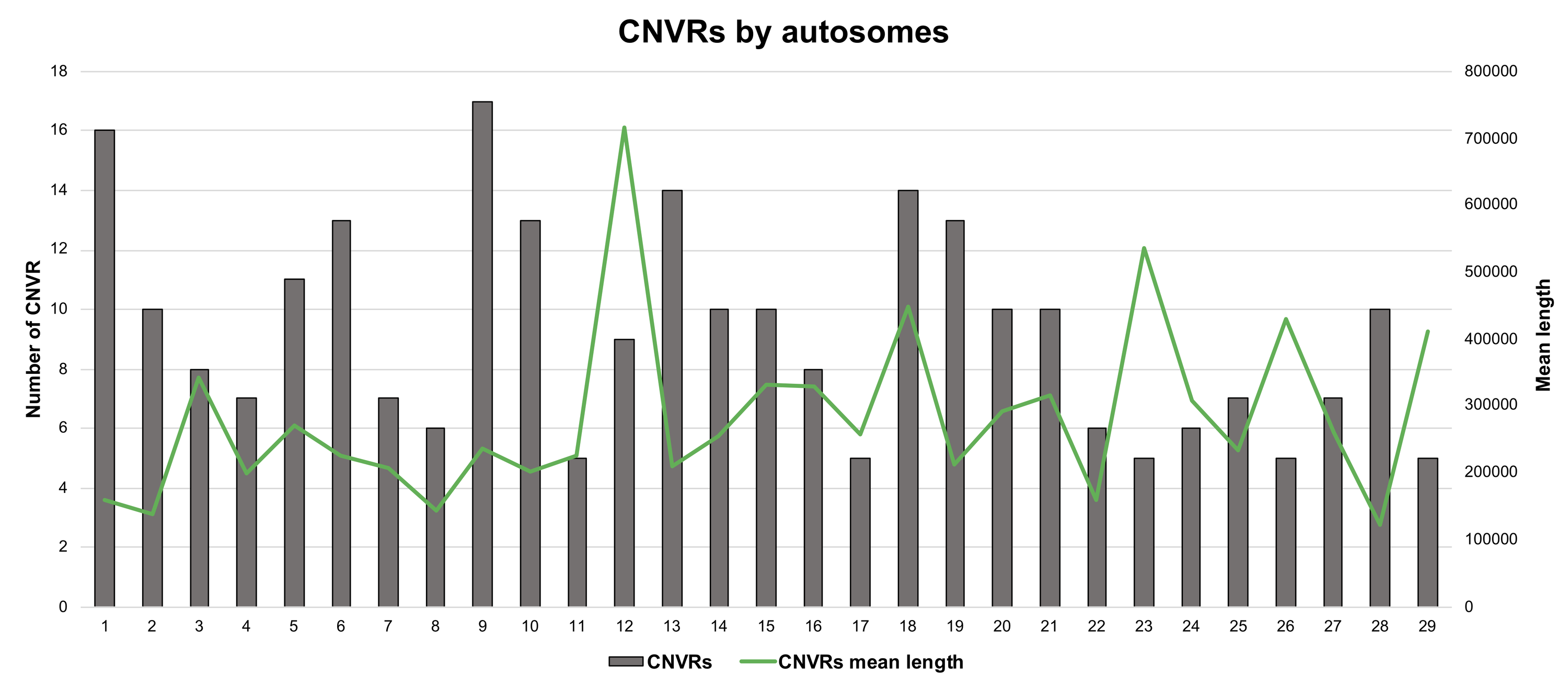

Supplement: S1 Fig — (TIF) [file pone.0303044.s005.tif]

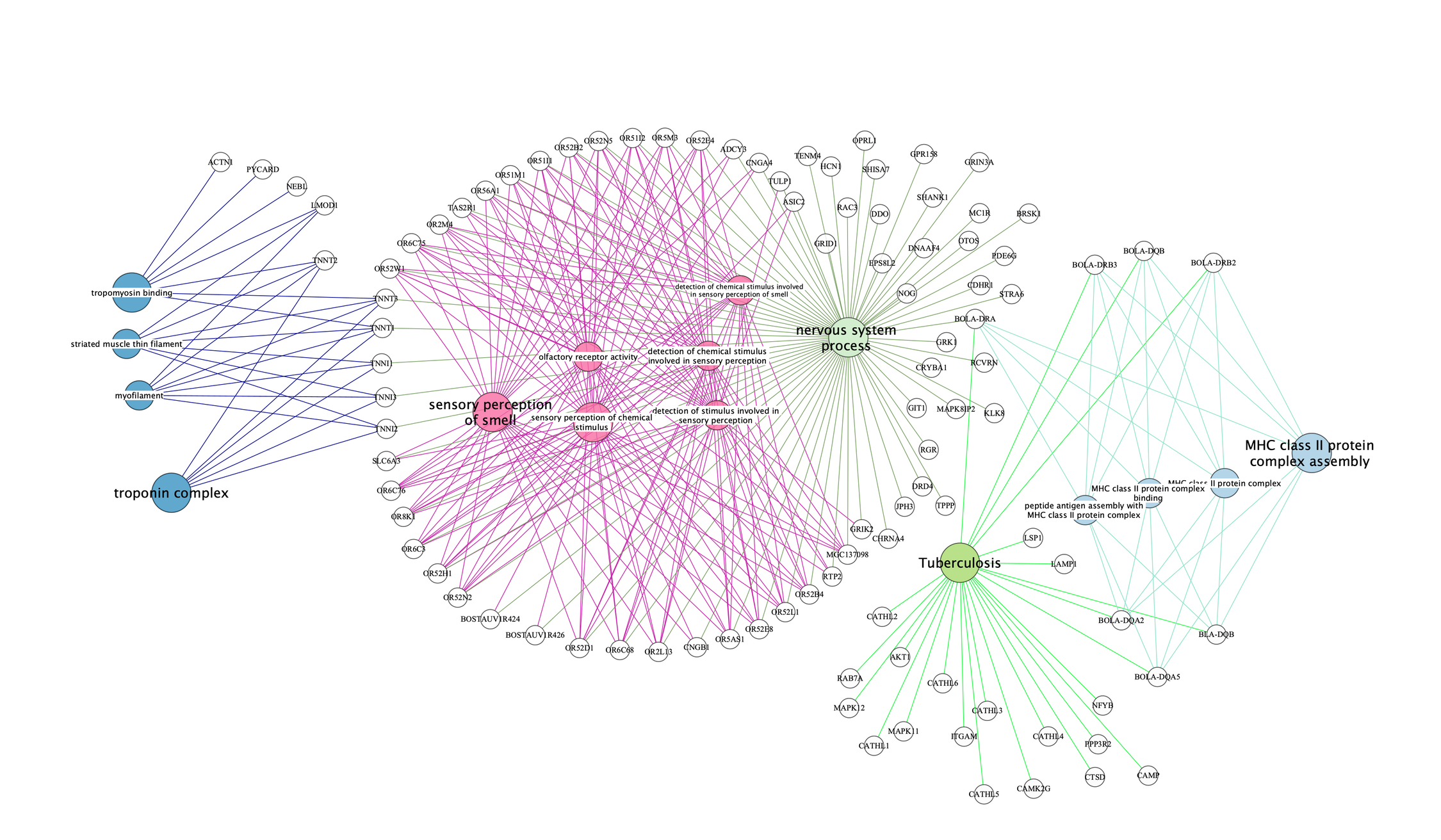

Supplement: S2 Fig — (TIF) [file pone.0303044.s006.tif]
